# Effect of five-tone music-based intervention combined with the Yarward YH-997S nursing communication information system on psychological coping among family caregivers of patients with perioperative stroke

**DOI:** 10.3389/fpsyt.2026.1711024

**Published:** 2026-03-31

**Authors:** Kaifeng Yao, Xiangyang Zhu, Xiaoxiao Zhu, Lihua Wang

**Affiliations:** 1 Department of Gastrointestinal surgery, Affiliated Hospital No.2 of Nantong University, Nantong, China; 2 Department of Neurosurgery, Affiliated Hospital No.2 of Nantong University, Nantong, China; 3 Department of Nursing, Affiliated Hospital No.2 of Nantong University, Nantong, China

**Keywords:** family caregivers, five-tone music-based intervention, nursing information system, perioperative psychological intervention, stroke

## Abstract

**Objective:**

Family caregivers of patients with perioperative stroke frequently experience elevated levels of psychological distress. This study assessed the effects of combining five-tone music-based intervention with the Yarward YH-997S Communication Information Nursing System on reducing symptoms of anxiety and depression and enhancing psychological coping strategies.

**Methods:**

In this randomized controlled trial, a total of 120 family caregivers of patients with perioperative stroke were randomly assigned to either an intervention group (n = 60) or a control group (n = 60). Both groups received routine nursing support—standard caregiver education and communication from nursing staff. The intervention group additionally received five-tone music-based intervention combined with the Yarward YH-997S Communication Information Nursing System for 10 consecutive days. Psychological status was assessed using the Self-Rating Anxiety Scale (SAS), Self-Rating Depression Scale (SDS), and Coping Inventory for Stressful Situations, administered prior to and following the intervention.

**Results:**

Compared to the control group, the intervention group demonstrated significantly lower SAS and SDS scores after the intervention. Task-oriented coping scores increased, whereas emotion-oriented and avoidance-oriented coping scores decreased. Psychological well-being improved significantly in the intervention group. Furthermore, nursing satisfaction and compliance with the intervention were higher in the intervention group than in the control group, with statistically significant differences.

**Conclusion:**

Combining five-tone music-based intervention with the Yarward YH-997S Communication Information Nursing System effectively alleviated negative emotional states, enhanced psychological adaptability, and optimized coping strategies among family caregivers of patients with perioperative stroke. These findings indicate that this combined approach represents a feasible and sustainable nursing intervention.

## 1 Introduction

Stroke exhibits a high incidence among middle-aged and older adults, characterized by sudden onset, rapid progression, and a high rate of disability. These clinical features often necessitate complex surgical procedures and place substantial demands on perioperative nursing management (1). During this period, family caregivers often assume considerable caregiving responsibilities and are susceptible to psychological distress, including symptoms of anxiety, depression, and insomnia. These factors can negatively influence adherence to treatment regimens and hinder the effective implementation of nursing care. Elevated caregiver stress levels can also compromise caregiver well-being, disrupt communication between healthcare providers and patients, and contribute to reduced satisfaction with nursing services (2).

Within multidisciplinary care models, non-pharmacological psychological interventions have gained recognition for their accessibility and low-risk profile. Traditional Chinese five-tone music-based intervention, supported by both classical theory and emerging clinical evidence, has demonstrated potential in promoting emotional regulation and psychological relaxation. “Qi” in traditional Chinese medicine refers to vital life energy responsible for driving blood circulation, maintaining physiological balance, and regulating bodily functions. Harmonization of Qi is believed to support the functional balance of the five zang organs and contribute to overall health. By applying distinct tonal modalities corresponding to each zang organ, five-tone music -based intervention aims to regulate Qi and blood, relieve emotional stagnation, and ultimately enhance psychosomatic well-being (3). In parallel, nursing data platforms provide caregivers with continuous and stable informational support by improving the efficiency of knowledge acquisition and facilitating interactive communication (4). The Yarward YH-997S Nursing Communication Information System is an integrated communication and information management solution specifically designed for modern medical institutions. Its core objective is to optimize the workflow of medical staff, enhance real-time connection between patients and medical personnel, and thereby improve the efficiency of nursing and the quality of service.

The system mainly consists of the following hardware components:

1. Bedside terminal: Located beside each hospital bed, it enables patients to make calls, engage in intercom conversations, control indoor lighting and entertainment systems.
2. Nurse workstation: The core management device in the nurse station, it is used to receive, display and process all call requests from wards, and to monitor the status and alert information of all patients in the hospital in real time.
3. Door display: Installed outside each ward, it shows the room status (such as “Under Surgery”, “Do Not Disturb”) and the information of the responsible nurse.
4. Wearable receiver: A portable receiving device (such as a watch-type receiver) worn by nurses, ensuring they can receive call alerts in real time even when on the move.

Through the collaborative operation of these components, the system realizes functions such as call management, information boards, nursing scheduling and data statistics, and is an important part of building smart wards and digital hospitals. The Yarward YH-997S Communication Information Nursing System, a smart nursing management platform, enables personalized delivery of nursing information, incorporates multimedia support for psychological interventions, and facilitates seamless communication among patients, caregivers, and healthcare professionals.

The development of a scientific, systematic, and user-friendly psychological support framework is essential for mitigating the emotional burden experienced by family caregivers during the perioperative period, while improving the structure and satisfaction of nursing services. Integrating five-tone music-based intervention with the Yarward YH-997S Communication Information Nursing System may enhance the humanistic dimension of perioperative stroke care, providing a novel model that combines traditional Chinese therapeutic principles with digital nursing technologies. Accordingly, this study aimed to evaluate the effects of this integrated intervention on anxiety, depression, and psychological coping among family caregivers of perioperative stroke patients. We hypothesized that, compared with routine perioperative care, the combined application of five-tone music-based intervention and the Yarward YH-997S Communication Information Nursing System would reduce negative emotional symptoms and enhance adaptive psychological coping strategies.

## 2 Materials and methods

### 2.1 Participants and eligibility criteria

A total of 120 family caregivers of patients with stroke who were undergoing perioperative treatment in the Department of Neurosurgery at our hospital were recruited between June 2023 and June 2024. All participants were primary caregivers of patients with stroke, were in generally good health, demonstrated the ability to understand study-related information, agreed to adhere to the study protocol, and provided written informed consent. The consent form explicitly stated that the intervention involved playing a specific audio track (Mu Yang Qu) through the Yarward YH-997S Communication Information Nursing System and authorized the research team to collect and analyze anonymized digital data related to the intervention (e.g., assessment scores, usage logs) for research purposes. No audio or video recordings of the participants themselves were made in this study.

The inclusion criteria were: (1) age ≥ 18 years, with no restriction on sex or educational background; (2) family caregivers of patients admitted for their first stroke surgery and scheduled for surgical treatment; (3) serving as the primary caregivers with a minimum daily caregiving time of ≥ 8 hours; and (4) possessing adequate cognitive and communication abilities who were able to use smart devices for data collection. The threshold of ≥ 8 hours was set to ensure the rigorous selection of primary caregivers who bear the main caregiving responsibility and have sufficient interaction with the patient. This criterion was intended to ensure that the intervention could be effectively implemented and that the caregivers’ psychological stress process could be fully observed.

The exclusion criteria were: (1) family caregivers with severe mental or neurological disorders; (2) participation in other psychological intervention studies during the same period; and (3) strong aversion to music or the presence of hearing impairments.

Participation was voluntary, and the study protocol was reviewed and approved by the ethics committee of the hospital.

**Figure 1:**
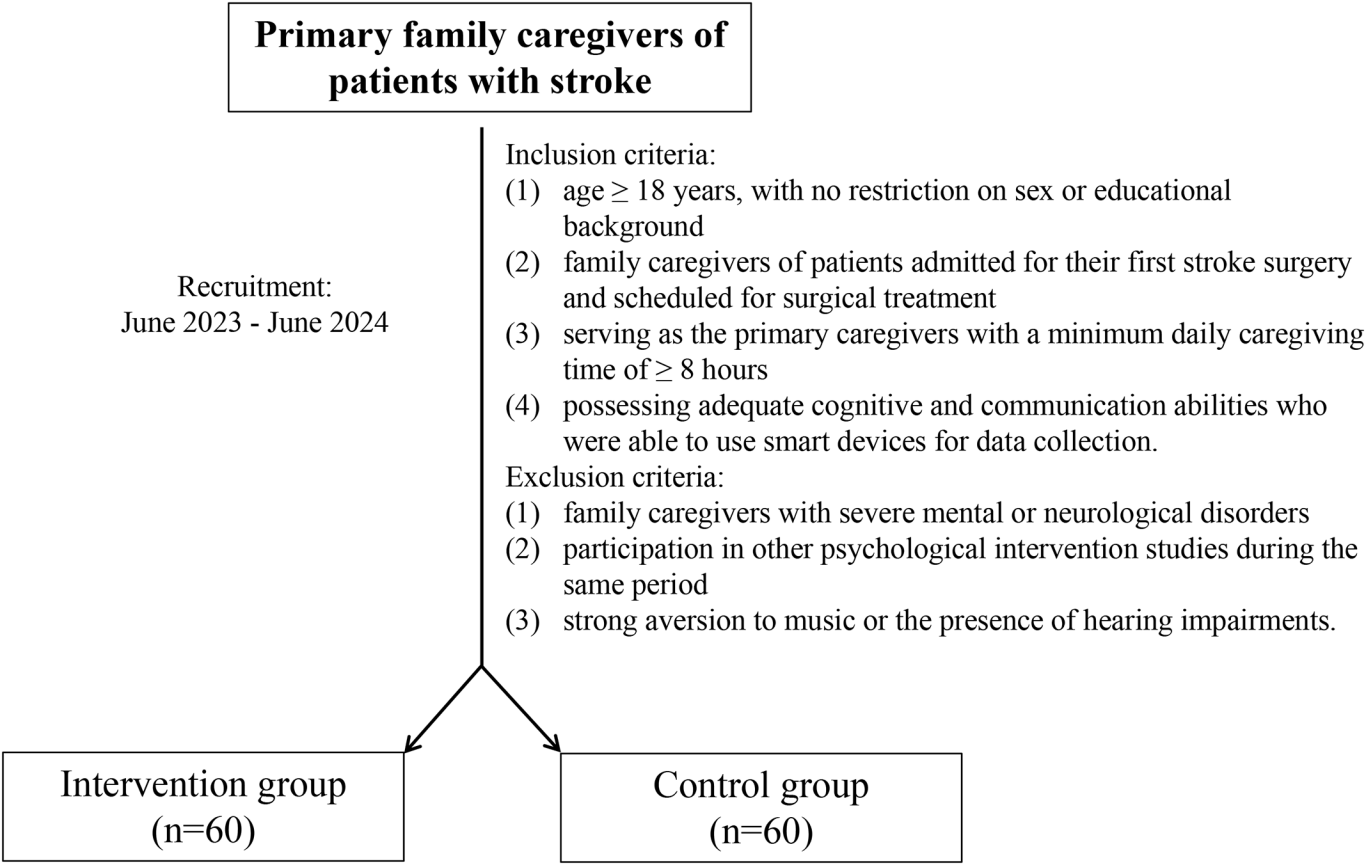
Study flowchart.

The study flowchart is shown in **Figure 1**.

### 2.2 Methods

A randomized controlled trial design was used to ensure scientific rigor and reproducibility of the intervention effects. A total of 120 primary family caregivers of patients with stroke were randomly assigned in a 1:1 ratio to the intervention group (n = 60) or the control group (n = 60). Randomization was conducted using a random-number table, and sequential numbers were allocated immediately after enrollment. The allocation process was carried out independently by a third party to reduce subjective assignment bias. Statistical analysis demonstrated no significant differences between the two groups in terms of age, sex, educational level, caregiving time, or relationship to the patient (all *p* > 0.05), confirming good baseline comparability.

#### 2.2.1 Control group

Family caregivers in the control group received routine nursing support—standard caregiver education and communication from nursing staff. Prior to surgery, the charge nurse provided explanations of surgical procedures, early-warning signs of postoperative complications, key precautions, and basic rehabilitation knowledge, thereby establishing a foundational level of understanding for caregivers. During the intraoperative period, caregivers were informed of patient condition updates and changes to medical orders, with the aim of reducing uncertainty-related anxiety. In the early postoperative stage, caregivers were given instructions on basic care and reassurance to facilitate appropriate expectations regarding recovery.

These interactions did not involve structured psychological interventions or the use of a digital nursing platform. Communication was limited to face-to-face exchanges and feedback derived from patient observation. The duty nurse documented the content of caregiver interactions and noted observable changes in caregiver emotional state.

#### 2.2.2 Intervention group

Family caregivers received routine nursing support, including standard caregiver education and communication from nursing staff, as in the control group. In addition, caregivers in the intervention group participated in a combined program of five-tone music-based intervention and the Yarward YH-997S Communication Information Nursing System, providing both emotional regulation and informational support. The intervention lasted 10 days, beginning three days before surgery and continuing until the seventh postoperative day. This design was based on the need to comprehensively cover the key phases of perioperative psychological stress, as family caregivers of stroke patients experience distinct patterns of anxiety, decision-making pressure, and coping demands before and after surgery, and short-term, intensive psychological interventions (e.g., delivered multiple times per week) have been reported to yield positive effects on emotional outcomes. Five-tone music-based intervention demonstrated measurable benefits in patients with post-stroke depression within 1–2 weeks; therefore, we positioned this short-term, intensive intervention within the acute stress window to provide timely and targeted psychological support. Activities were supervised jointly by charge nurses and study investigators.

Music-based intervention protocol: Developed by a certified traditional Chinese medicine (TCM) music-based intervention team, the protocol began with an emotional assessment to identify predominant emotional states, matched with corresponding tonal therapy: Gong tone (宫音) for the spleen, which governs thinking; the Shang tone (商音) for the lungs, which govern grief; the Jue tone (角音) to disperse liver Qi stagnation, which governs anger; the Zhi tone (徵音) to invigorate heart Qi, which governs joy; and the Yu tone (羽音) to calm the kidneys, which govern fear (5).

All musical selections in this study strictly followed the Traditional Chinese Medicine (TCM) “Five-Tone Healing” theory, in which the five tones (i.e. Gong, Shang, Jue, Zhi, and Yu) correspond to the five viscera. Considering the common perioperative emotional states of family members of stroke patients, particularly anxiety and excessive worry, we primarily selected Gong-mode music, which is associated with the spleen and is traditionally believed to calm the mind. Specifically, the piece “Mu Yang Qu (Shepherd’s Song)” was chosen for its purported spleen-fortifying and anxiety-relieving effects. “Mu Yang Qu” was composed by Mr. Wang Liping. The audio files were downloaded from QQ Music, selected from the “Xi Liang Bao – Wellness Music Collection,” featuring traditional Chinese instruments such as the dizi (bamboo flute), xiao, and guzheng performing this classic piece. The track duration is 10 minutes and was played on loop three times per session, resulting in a 30-minute intervention. The music was delivered once daily through the Yarward YH-997S Nursing Communication Information System.

Music sessions were held twice daily at 10:00 AM and 4:00 PM, each lasting 30 minutes, delivered through specialized headphones. Smartphones were not permitted during the intervention. All participants received a standardized music intervention by listening to the fixed track Mu Yang Qu. Emotional assessments during the intervention were used to monitor participants’ overall receptivity and general emotional responses, not to personalize the intervention content.

Yarward YH-997S Communication Information Nursing System intervention: Caregivers registered in the Yarward YH-997S Communication Information Nursing System platform and were enrolled in a patient-specific perioperative nursing plan. The system provided daily perioperative knowledge modules, which included content on the pathological mechanisms of stroke, surgical risks, postoperative nutritional management, and functional exercise procedures.

An emotional management module was incorporated, requiring family caregivers to complete a daily five-item emotional self-rating, which automatically generated tailored recommendations (e.g., specific tonal music, mindfulness audio, or breathing exercise videos). A messaging function allowed family caregivers to submit inquiries, which were addressed collectively by charge nurses, and video consultations were held every 48 hours. In the Yarward nursing information system, family caregivers’ feedback was addressed through a combination of asynchronous messaging and scheduled video consultations. Specifically, caregivers submitted questions via the system’s message board, which were reviewed and answered within 8 hours by the responsible nursing team under the supervision of the head nurse. In addition, the head nurse chaired a weekly group video conference to discuss common concerns and complex cases. This integrated approach ensured both timely responses and in-depth communication, with clear accountability assigned to a head nurse–led responsible nursing team.

The system automatically tracked login frequency, reading progress, and emotional score fluctuations, providing objective measures of adherence.

The dual-path parallel management model in the intervention group was guided by the principles of active identification, personalized matching, and real-time feedback. The emotion-regulating effects of music-based intervention were integrated with the cognitive and informational support provided through the Yarward YH-997S Communication Information Nursing System, forming a structured and iterative intervention process. All activities were standardized under a unified protocol, delivered by nursing staff trained and certified in the procedures, and monitored by research specialists to ensure accuracy of data collection and compliance with study requirements.

All participating family members listened to the same pre-selected piece during the intervention period. While emotional assessments were used to monitor participants’ responses, the musical content itself remained consistent to allow preliminary evaluation of the overall effect of this standardized protocol.

**Figure 2:**
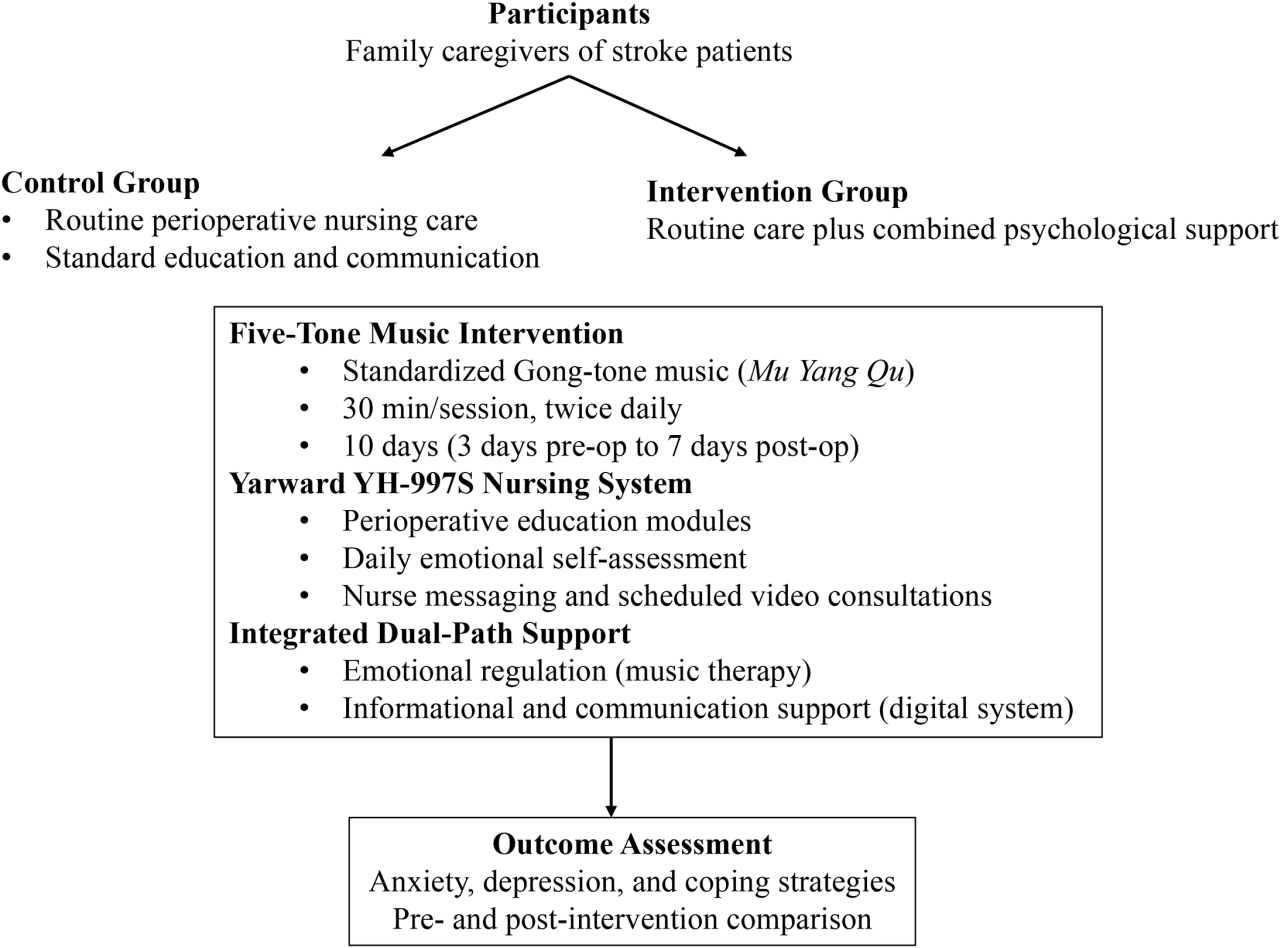
The intervention procedure.

The intervention procedure is presented in **Figure 2**.

### 2.3 Data acquisition

#### 2.3.1 Measurement instruments

Self-rating anxiety scale (SAS): The SAS, developed in 1971 by American psychologist William Zung, is one of the most widely applied tools for assessing anxiety in both clinical and research settings. In this study, the validated Chinese version of the SAS was used to assess anxiety levels among family caregivers of patients with perioperative stroke (6). The scale consists of 20 items designed to capture subjective anxiety experiences and associated somatic symptoms, including palpitations, restlessness, irritability, and fatigue within the preceding week. Each item is rated on a four-point Likert scale (1 = none or almost none of the time; 4 = most or all the time). The total score is multiplied by 1.25 to generate a standard score, with scores above 50 indicating the presence of anxiety symptoms.

Self-rating depression scale (SDS): The SDS developed by Zung, is a standardized instrument for assessing mild to moderate depressive states and is widely applied in quantitative studies within psychological nursing and mental health research. In this study, the Chinese version of the SDS certified by the Chinese Psychological Society was used (7). The scale consists of 20 items across 4 dimensions: emotional, somatic, cognitive, and behavioral, covering symptoms like depressed mood, loss of interest, sleep disturbances, and reduced energy. Each item is scored on a four-point frequency scale, with higher scores indicating greater severity of depressive symptoms. The total score is multiplied by 1.25 to yield a standard score. A standard score ≥ 53 indicates the presence of depressive symptoms, with scores of 53–62 representing mild, 63–72 moderate, and ≥ 73 severe depression.

Coping inventory for stressful situations (CISS): The Chinese version of the CISS, translated and culturally adapted, was employed (8). The scale consists of 48 items that assess 3 primary coping styles: task-oriented coping, emotion-oriented coping, and avoidance-oriented coping. Task-oriented coping emphasizes rational problem analysis and solution-seeking, emotion-oriented coping involves increased attention to emotional experiences like anxiety and anger, and avoidance-oriented coping encompasses behavioral escape and cognitive denial. The Chinese version of this instrument has demonstrated good reliability and validity in Chinese populations, with Cronbach’s α coefficients ranging from 0.66 to 0.81 across its dimensions.

#### 2.3.2 Data collection procedures

All scales were administered in their standard Chinese versions. Uniform instructions were provided by a designated investigator to guide family caregivers in completing the assessments. Evaluations were conducted one day preoperatively and seven days postoperatively. Data accuracy and completeness were ensured through double-checking by two investigators, and the entire data acquisition process was supervised by professional data management personnel.

All paper questionnaires were numbered and archived by designated personnel, followed by double entry using EpiData 3.1 to ensure accuracy of data entry. Regular intervention feedback meetings were convened every three days to summarize intervention progress, caregiver satisfaction, and special circumstances. Resources were promptly allocated by the principal investigator to address emergent issues. To reduce research bias, all assessors were blinded to participant allocation prior to scoring, implementing a single-blind design. Furthermore, a data desensitization mechanism was applied, with caregiver data processed using unique codes to strictly control the risk of privacy disclosure.

### 2.4 Statistical analysis

All data were analyzed using SPSS 26.0 statistical software. The dataset was examined for completeness and logical consistency, and data entry accuracy was ensured through a dual-entry method conducted by two independent investigators. Quantitative variables, including SAS, SDS, and CISS scores, were first tested for normality using the Shapiro–Wilk test. Normally distributed data are presented as mean ± standard deviation. For these variables, intergroup comparisons were conducted using independent-samples *t* tests, and intragroup pre–post comparisons were performed using paired *t* tests. For data that did not conform to a normal distribution, non-parametric tests such as the Mann–Whitney U test or the Wilcoxon signed-rank test were applied. Categorical variables are summarized as frequencies and percentages, with intergroup differences assessed using the chi-squared (χ²) test or Fisher’s exact test as appropriate. Correlation analyses were conducted using Pearson’s correlation coefficient for normally distributed continuous variables and Spearman’s rank correlation coefficient for non-normal or ordinal data. All statistical tests were two-sided, with *p* < 0.05 considered statistically significant. Effect sizes were calculated where applicable to supplement the interpretation of statistical results.

A *post hoc* power analysis was conducted to assess the statistical power of the tests. Based on the observed post-intervention outcomes, the effect sizes (Cohen’s *d*) for both anxiety (SAS) and depression (SDS) scores between the two groups were approximately 1.12, indicating a very large effect. Using this effect size, a significance level of α = 0.05, and a sample size of 60 participants per group, *post hoc* analysis yielded a statistical power greater than 0.999. This value far exceeds the conventional threshold of 0.80, indicating that the current sample size was more than sufficient and had excellent sensitivity to detect true intervention-related differences.

## 3 Results

### 3.1 Comparison of general data

No statistically significant differences were observed between the control and intervention groups in demographic characteristics, educational level, caregiving duration, kinship, or prior experience with psychological counseling (all *p* > 0.05), demonstrating good baseline comparability. The comparison of baseline characteristics between the groups is depicted in **Table 1**.

**Table 1:** Comparison of general demographic characteristics between intervention and control groups.

Item	Intervention (*n* = 60)	Control (*n* = 60)	*P*-value
Mean age (year)	46.8 ± 11.4	47.3 ± 10.9	0.763
Sex (male/female)	27 / 33	29 / 31	0.714
Marital status (married/%)	54 (90.0%)	56 (93.3%)	0.505
Education level (high school and below/bachelor's degree and above)	35 / 25	37 / 23	0.707
Occupation (healthcare/non-healthcare providers)	8 / 52	7 / 53	0.779
Relationship with patient (spouse/child/other)	22 / 32 / 6	25 / 30 / 5	0.866
Caregiving duration (hour/day)	10.5 ± 2.1	10.2 ± 2.3	0.432
Previous caregiving experience (yes/no)	21 / 39	18 / 42	0.545
History of psychological counseling (yes/no)	5 / 55	6 / 54	0.752
Family history of chronic diseases (yes/no)	9 / 51	10 / 50	0.798
Residence (urban/rural)	38 / 22	40 / 20	0.702
Monthly household income (RMB)	6120 ± 1320	5980 ± 1410	0.456
Religious beliefs (yes/no)	7 / 53	6 / 54	0.765
Use of smartphone (yes/no)	60 / 0	60 / 0	–
Knowledge of five-tone music therapy (yes/no)	3 / 57	2 / 58	0.647

For these variables, intergroup comparisons were conducted using independent-samples *t* tests, and intragroup pre–post comparisons were performed using paired *t* tests. For data that did not conform to a normal distribution, non-parametric tests such as the Mann–Whitney U test or the Wilcoxon signed-rank test were applied. Categorical variables are summarized as frequencies and percentages, with intergroup differences assessed using the chi-squared (χ²) test or Fisher’s exact test as appropriate.

### 3.2 Comparison of SAS and SDS scores prior to intervention

Prior to the intervention, no significant differences were observed in SAS and SDS scores between the two groups of family caregivers, indicating comparable baseline psychological states. The SAS scores of both groups were within the mild-to-moderate anxiety range, while the SDS scores reflected a tendency toward mild depression, consistent with the elevated psychological stress typically experienced by family caregivers during the perioperative period. Detailed results are presented in **Table 2**.

**Table 2:** Baseline SAS and SDS scores of family caregivers in the two groups ( $\overset{¯}{x}$ ± s).

Item	Intervention (*n* = 60)	Control (*n* = 60)	*t*-value	*p*-value
SAS score	58.4 ± 7.2	59.2 ± 6.8	-0.638	0.525
SDS score	55.6 ± 6.9	56.4 ± 7.1	-0.608	0.544

paired *t* test was applied.

### 3.3 Comparison of psychological status scores between groups after intervention

On day 7 of the intervention, SAS and SDS scores of family caregivers in the intervention group were significantly lower than those in the control group (*p* < 0.01), demonstrating that five-tone music-based intervention combined with the Yarward YH-997S Communication Information Nursing System effectively alleviated anxiety and depression during the perioperative period. The reduction rates of SAS and SDS scores in the intervention group were 34.7% and 36.5%, respectively, which were significantly higher than those observed in the control group (19.4% and 24.4%, respectively). Detailed results are presented in **Table 3**.

**Table 3:** Comparison of SAS and SDS scores between the two groups after intervention ( $\overset{¯}{x}$ ± s).

Item	Intervention (*n* = 60)	Control (*n* = 60)	*t*-value	*p*-value
SAS score	38.1 ± 6.5	45.7 ± 7.1	-6.038	<0.001
SDS score	35.3 ± 6.2	42.6 ± 6.8	-5.785	<0.001

paired *t* test was applied.

### 3.4 Comparison of CISS coping style scores after intervention

Results of the CISS demonstrated a significant increase in task-oriented coping scores in the intervention group, accompanied by significant decreases in emotion-oriented and avoidance-oriented coping scores. These findings indicated a shift in coping styles from negative emotion-oriented patterns to positive task-oriented patterns among family caregivers. In contrast, no significant changes were observed in the control group, with slight increases in emotion-oriented and avoidance-oriented coping noted in some participants, reflecting limited psychological adjustment under routine nursing support. Detailed results are presented in **Table 4**.

**Table 4:** Comparison of CISS coping style scores between the two groups after intervention ( $\overset{¯}{x}$ ± s).

Items	Intervention (*n* = 60)	Control (*n* = 60)	*t*-value	*p*-value
Task-oriented coping	68.4 ± 6.9	59.6 ± 7.5	6.703	<0.001
Emotion-oriented coping	39.1 ± 5.8	46.5 ± 6.2	-6.516	<0.001
Avoidance-oriented coping	35.4 ± 5.6	42.3 ± 6.0	-6.390	<0.001

paired *t* test was applied.

### 3.5 Intervention compliance and satisfaction of family caregivers

High compliance was maintained by family caregivers in the intervention group throughout the intervention period, with a music playback completion rate of 96.7%. The Yarward YH-997S Communication Information Nursing System was accessed an average of 2.8 times per day, and the content reading completion rate exceeded 90%. In the satisfaction survey conducted 7 days postoperatively, 93.3% of family caregivers in the intervention group reported being “very satisfied” or “satisfied,” a significantly higher proportion than that observed in the control group (70.0%). Scores in the intervention group were significantly higher than those in the control group for nursing communication, emotional support, and knowledge acquisition, demonstrating that the combined intervention contributed to improvements in psychological status and the overall caregiving experience.

## 4 Discussion

The significantly lower SAS and SDS scores observed in the intervention group indicate that the combination of five-tone music-based intervention and the Yarward YH-997S Communication Information Nursing System effectively alleviated anxiety and depression among family caregivers of patients with perioperative stroke. Caregivers in this setting are exposed to sustained phycological stress due to clinical uncertainty, decision-making pressure, and the anticipation of long-term caregiving demands. Previous studies have shown that routine nursing support, which mainly relies on verbal reassurance and postoperative explanations, is often insufficient to address the persistent psychological burden experienced by caregivers because it lacks structured emotional and cognitive regulation strategies (9, 10). Our findings suggest that a more systematic intervention addressing both emotional and informational needs can produce more stable psychological benefits.

The therapeutic mechanism underlying five-tone music intervention can be understood from both TCM theory and modern neurophysiological perspectives. The tonal frequencies corresponding to the five zang-organs are believed to regulate emotional balance in TCM, while contemporary studies indicate that music therapy modulates autonomic nervous system activity and promotes the release of endogenous calming mediators, thereby reducing anxiety and improving emotional stability. In this study, individualized tonal music was applied to improve the suitability and acceptability of emotional regulation. The Yarward YH-997S Communication Information Nursing System provided cognitive support by enabling family caregivers to form reasonable expectations, acquire scientific nursing knowledge, and reduce anxiety related to uncertainty through frequent information delivery, daily self-assessments, and real-time communication.

The combined approach addressed both emotional regulation and cognitive support, creating an integrated psychological intervention framework that demonstrated clear advantages over conventional nurse-led, unidirectional education. Our study found that caregivers in the intervention group showed a significant increase in scores on the trust-oriented coping dimension, reflecting an internal shift toward more positive, task-oriented coping strategies. This can be interpreted through a cognitive–emotional dual-pathway model. Specifically, the Yarward nursing system provides continuous and transparent informational support, which directly enhances caregivers’ sense of cognitive control and predictability within the medical environment, thereby laying a rational foundation for trust. Meanwhile, five-tone therapeutic music exerts an emotion-regulating effect that alleviates anxiety, helplessness, and other negative emotions, reduces emotional barriers, and facilitates the development of affective trust. Through this synergistic interaction, caregivers are guided away from passive, emotion-driven coping patterns toward more active, trust-based and collaborative coping modes. This finding is consistent with Paterick et al., who emphasized communication and information as cornerstones of medical trust (11). Our results further suggest that combining structured informational support with culturally resonant, non-pharmacological emotional regulation strategies can more systematically foster trust and, in turn, more effectively promote positive coping behaviors. The perioperative phase of stroke care is characterized by fluctuating clinical status and frequent high-risk events, which impose continuous psychological strain on family caregivers. Without targeted support, maladaptive coping behaviors, such as worry, withdrawal, or avoidance of communication, may emerge, intensifying caregiver distress and impeding effective care coordination. The interventions applied in this study stabilized the emotional states of family caregivers and promoted proactive cognitive engagement through structured information delivery and responsive feedback mechanisms. The Yarward YH-997S Communication Information Nursing System reinforced behavioral consistency via daily educational modules and emotional feedback functions, positioning caregivers as active participants in perioperative management. Music-based intervention enhanced emotional awareness and willingness for self-regulation, further reducing maladaptive stress responses and reinforcing psychological resilience (12).

This shift in coping style contributed to improved psychological well-being of family caregivers and facilitated more effective caregiving behaviors, which were associated with higher patient adherence to nursing instructions and improved postoperative recovery trajectories.

The dual-intervention “music + platform” model developed in this study illustrates a pathway that combines technological efficiency with cultural sensitivity, offering a novel approach for intelligent nursing practice. Traditional nursing practice is often constrained by high patient volumes and limited communication time, while family members may experience anxiety or distrust due to inadequate information transparency and delayed feedback. The Yarward YH-997S Communication Information Nursing System reduced these constraints and enhanced communication efficiency between caregivers and nursing staff. Features such as multimedia knowledge modules, voice prompts, and emotional monitoring facilitated the establishment of standardized yet personalized nursing support (13).

Simultaneously, five-tone music-based intervention, as a representative non-pharmacological intervention in TCM, incorporated an element of humanistic care into the intervention strategy. Beyond its emotional regulatory effects, the therapy provided cultural resonance and increased acceptability among caregivers (14, 15). In this study, caregiver satisfaction with the intervention reflected the practical value of integrating music-based intervention with digital support platforms. This model offers a feasible strategy for strengthening family caregiver support systems in high-stress clinical settings such as neurosurgery and intensive care, while providing a foundation for incorporating advanced technologies such as artificial intelligence and big data into humanistic nursing services. The high caregiver satisfaction observed in this study reflects the practical value of combining technological efficiency with culturally sensitive care.

Compared with purely technology-assisted interventions, such as the SINEMA program (16), which primarily empowers village physicians and focuses on patient management, or the TASK III program (17), which provides caregivers with remote skills training, the combined intervention of five-tone music therapy plus the Yarward system adopted in this study is fundamentally different. It goes beyond the provision of informational or technical support by explicitly incorporating a culturally resonant, non-pharmacological approach to emotional regulation. As a result, the intervention extends from a predominantly cognitive–behavioral level to a deeper emotional–psychological level. This added dimension may explain its greater effectiveness in fostering caregivers’ internal sense of trust and promoting positive coping strategies, such as task-oriented coping, outcomes that are difficult to achieve through technical empowerment or educational support alone. Together, these findings suggest that integrating emotional, cognitive, and technological elements may represent a more comprehensive model for supporting family caregivers in high-stress clinical environments.

### 4.1 Limitations

This study has several limitations that should be considered. First, the sample size was relatively small (n = 120), which may limit the statistical power and generalizability of the findings. However, as an exploratory trial, the primary aim was to evaluate the feasibility and safety of the combined five-tone music-based intervention with the Yarward YH-997S Communication Information Nursing System intervention and to provide preliminary data for effect size estimation. Second, in this single-center randomized controlled trial, although random allocation was implemented, blinding of participants and nursing staff was not feasible due to the nature of the intervention, which may have introduced performance bias. Third, the study did not collect or control for key patient-level clinical variables, such as stroke severity (e.g., NIHSS score), postoperative complications, or neurological recovery, which may independently influence family members’ psychological responses. Future studies should address these limitations by conducting rigorously designed randomized controlled trials, applying formal sample size estimation based on current effect sizes, incorporating blinding where feasible, and systematically monitoring and adjusting for patients’ clinical prognostic indicators. Specifically, caregivers could be stratified and randomized according to patients’ stroke severity (NIHSS score) or the location of key lesions to ensure baseline comparability between groups. In statistical analyses, these factors could be included as covariates in generalized linear models to adjust for their potential confounding effects on psychological outcomes, thereby allowing a more accurate assessment of the net effect of the intervention. Lastly, the music intervention in this study was limited to a fixed track, which facilitates standardization but does not allow for individualized adjustments based on participant characteristics or real-time emotional feedback. Future studies could explore personalized music interventions using more advanced systems. Despite these limitations, the results offer valuable preliminary insights into the potential effectiveness and safety of this combined intervention in clinical practice. Additionally, the cultural adaptability of the five-tone music-based intervention requires careful consideration. As our hospital is a tertiary institution integrating Chinese and Western medicine, family members generally demonstrate high acceptance of traditional Chinese cultural concepts, including the theoretical basis of the Five-Tone Healing system. However, when applied to populations without such a cultural background, challenges may arise due to differences in theoretical acceptance and musical preferences. Future studies should therefore explore integrating the Five-Tone Healing principles with contemporary music-based intervention approaches to develop more culturally adaptable and widely applicable intervention models.

## 5 Conclusion

This study demonstrated that the dual-path intervention model combining five-tone music-based intervention with the Yarward YH-997S Communication Information Nursing System significantly reduced anxiety and depression levels (SAS/SDS scores) among family caregivers of patients with perioperative stroke and facilitated a shift in coping styles toward active, task-oriented approaches. The key contributions of this model can be summarized as follows:

1. Physiological–cognitive synergistic intervention: Five-tone music-based intervention regulated neurotransmitter release through acoustic wave resonance, stabilizing the physiological basis of emotional regulation, while the Yarward YH-997S Communication Information Nursing System reinforced cognitive restructuring and alleviated anxiety related to informational uncertainty through frequent information delivery and interactive feedback.
2. Closed-loop support system: Integration of emotion-focused regulation with behavior-focused guidance established a bidirectional feedback loop, addressing the limitations of conventional unidirectional health education in routine nursing support.
3. Improvement in nursing quality: Enhanced psychological resilience among family caregivers contributed to improved patient adherence to nursing care, thereby accelerating postoperative rehabilitation.

Future research should focus on: (1) Advancing intelligent nursing integration through AI-driven emotion recognition and big data-based personalized recommendations; (2) exploring innovations in that combine non-pharmacological therapies from TCM with modern digital technologies to establish nursing paradigms characterized by both technological efficiency and cultural sensitivity; and (3) extending and validating this model in high-stress clinical settings such as intensive care and oncology to develop a standardized psychological support systems for family caregivers.

This study integrated a systematic nursing care process with culturally adapted audio interventions through a digital support platform, demonstrating a significant reduction in psychological stress among caregivers of stroke patients. The core innovation lies in the transferability of the framework: in the Chinese context, we incorporated the traditional Chinese medicine theory of the Five Tones for health; in global applications, the framework can be flexibly adapted to local culture and evidence, integrating indigenous music or mindfulness guidance to provide universally applicable caregiver support.

## Data Availability

The original contributions presented in the study are included in the article/supplementary material. Further inquiries can be directed to the corresponding author.
